# Advanced environmental scanning electron microscopy reveals natural surface nano-morphology of condensed mitotic chromosomes in their native state

**DOI:** 10.1038/s41598-024-63515-9

**Published:** 2024-06-06

**Authors:** Vilém Neděla, Eva Tihlaříková, Petr Cápal, Jaroslav Doležel

**Affiliations:** 1 Institute of Scientific Instruments of the Czech Academy of Sciences, Královopolská 147, Brno, 612 00 Czech Republic; 2 Institute of Experimental Botany of the Czech Academy of Sciences, Centre of Plant Structural and Functional Genomics, Šlechtitelů 31, Olomouc, 772 00 Czech Republic

**Keywords:** Scanning electron microscopy, Plant sciences

## Abstract

The challenge of in-situ handling and high-resolution low-dose imaging of intact, sensitive and wet samples in their native state at nanometer scale, including live samples is met by Advanced Environmental Scanning Electron Microscopy (A-ESEM). This new generation of ESEM utilises machine learning-based optimization of thermodynamic conditions with respect to sample specifics to employ a low temperature method and an ionization secondary electron detector with an electrostatic separator. A modified electron microscope was used, equipped with temperature, humidity and gas pressure sensors for in-situ and real-time monitoring of the sample. A transparent ultra-thin film of ionic liquid is used to increase thermal and electrical conductivity of the samples and to minimize sample damage by free radicals. To validate the power of the new method, we analyze condensed mitotic metaphase chromosomes to reveal new structural features of their perichromosomal layer, and the organization of chromatin fibers, not observed before by any microscopic technique. The ability to resolve nano-structural details of chromosomes using A-ESEM is validated by measuring gold nanoparticles with achievable resolution in the lower nanometre units.

## Introduction

The progress in biological research depends largely on new methods and instrumentation. In order to reveal details of subcellular structures and their dynamics, microscopic techniques with spatial resolution in nanometer range have been particularly useful^1^. To study hydrated cells and tissues in liquid, a Wet-SEM sample holder with a one or two (microfluidic flow cell) ultra-thin electron-transparent windows can be used to separate the sample from vacuum conditions in SEM^2^ or STEM^3^. The signal of backscattered (BSE) or transmitted (TE) electrons form a so-called Z-contrast with image resolution in tens of nm for SEM and ones of nm for STEM. Samples are typically fixed using aldehydes, which denature and cross-link cellular proteins, resulting in artefacts such as protein aggregates^4^. Standard immunogold techniques and heavy metal staining, usually by uranyl acetate or osmium tetroxide, are used to enhance Z-contrast^2^.

An alternative method is the environmental scanning electron microscopy (ESEM), which permits both static and dynamic in-situ observations and physical/chemical analysis of electrically non-conductive wet samples^5–8^. Low-energy secondary electrons, BSE and X-Ray photons can be detected and the sample size is not limited by the cell^5^. However, highly complex interplay of environmental imaging parameters such as the gas pressure, humidity, and temperature has given ESEM the reputation of being difficult to use, coupled with the challenges of precisely controlling thermodynamic conditions around wet samples. The imaging signal is often degraded due to electron beam scattering in the gas (usually water vapor) and image features are obscured by liquid layer covering the sample. While this may be mitigated by higher beam energy and current at reduced scan rate, it can result in radiation damage of the sample. Therefore, extremely environmentally- and radiation-sensitive samples are difficult or impossible to image at high resolution and without chemical treatment of their structure. To overcome these limitations, we have developed a new approach termed advanced environmental scanning electron microscopy (A-ESEM) for in-situ handling and high-resolution imaging of extremely sensitive structures. Here we demonstrate the power and potential of A-ESEM by analyzing condensed mitotic chromosomes in their native state at nanometer scale.

Although intensively studied since the first description by Walther Flemming in 1882^9^, molecular composition and three-dimensional topology of mitotic chromosomes remain obscure. The basic level of chromatin organization is undisputed: DNA molecule wounds around octamers of core histone proteins forming nucleosomes, which are interconnected by a short DNA linker and arranged into a linear 10 nm fiber^10^. Higher level of chromatin organization was presumed to stem from the interaction of linker histone H1 with adjacent nucleosomes, resulting in 30 nm fiber^11^. While the formation of the 30 nm fiber was observed in vitro, its existence in vivo was not confirmed by cryo-electron microscopy^12^ and cryo-electron tomography^13^. A similar conclusion was made after the analysis of the so-called Kuhn length^14^. Using the ChromEMT method, which combines electron microscopy tomography with a labelling method to enhance the contrast of DNA, it was established that chromatin fiber had a flexible diameter of 5–24 nm^15^. In a recent study, the size of chromatin fiber 11.6 nm was determined using helium ion microscopy imaging of tungsten coated barley mitotic chromosomes^16^. This raises a question on spatial organization of the nucleosome fiber in condensed chromosomes during cell division. While several models have been proposed^17^, growing evidence supports a model in which chromatin loops emanate radially from a centrally located helical scaffold^18,19^.

Compared to intensively studied spatial organization of chromatin, less attention was paid to other components of chromosome body, such as proteins, despite the fact that almost 900 proteins were identified in mitotic chromosomes of barley^20^. With a few exceptions, the spatial arrangement of chromosomal proteins and their function remain unknown. Importantly, a recent observation revealed that perichromosomal layer comprises over 30% of the entire chromosome volume and more than 33% of chromosome protein mass^21^. The layer was found to be involved in important processes linked to chromosome function^22^, with some of its molecular components identified^23^, and a model for mutual interaction of some of its components has been suggested^24^. However, the three-dimensional organization of the chromosome surface is not known and detailed visualization in its native state was beyond the reach of any imaging technique.

Current knowledge of chromosome nanostructure derives mostly from the observations by scanning electron microscopy (SEM). Since SEM requires high vacuum to prevent electron scattering, the samples must be stabilized by chemical fixation, metal coating and dehydration^25^, which may cause undefined changes of the sensitive surface organization. ESEM may offer a way to address these challenges^6^ as wet samples are observed under high water vapor pressure (tens to thousands of Pa) and low sample temperature (typically a few degrees Celsius), thus preventing sample drying. The inability to directly determine thermodynamic parameters on the sample surface requires laborious protocol optimization to maintain a thin layer of liquid. However, the fluid covering the sample is masking the surface nanostructure and is the source of free radicals produced by irradiating beam^26^. In addition, beam scattering in the gas or liquid reduces signal-to-noise ratio and may compromise image resolution.

Here we introduce an innovative approach solving the fundamental problem of the incompatibility of electron microscopy with the presence of liquid in any specimen. It is suitable for imaging of wet/live samples or high-resolution imaging of extremely sensitive nano-structures and nano-surfaces. In contrast to previous SEM studies and conventional ESEM, our method avoids the liquid layer covering the sample surface, stabilizes the wet sample in a thermodynamic equilibrium, enables high-resolution and low-dose irradiation imaging, suppresses radiation damage, eliminates the need for cryo-SEM sample preparation and simplifies the operation of the microscope with a vision of fully automatic control of parameters by artificial intelligence software.

## Results and discussion

### Development of the artificial intelligence assisted A-ESEM

**Figure 1:**
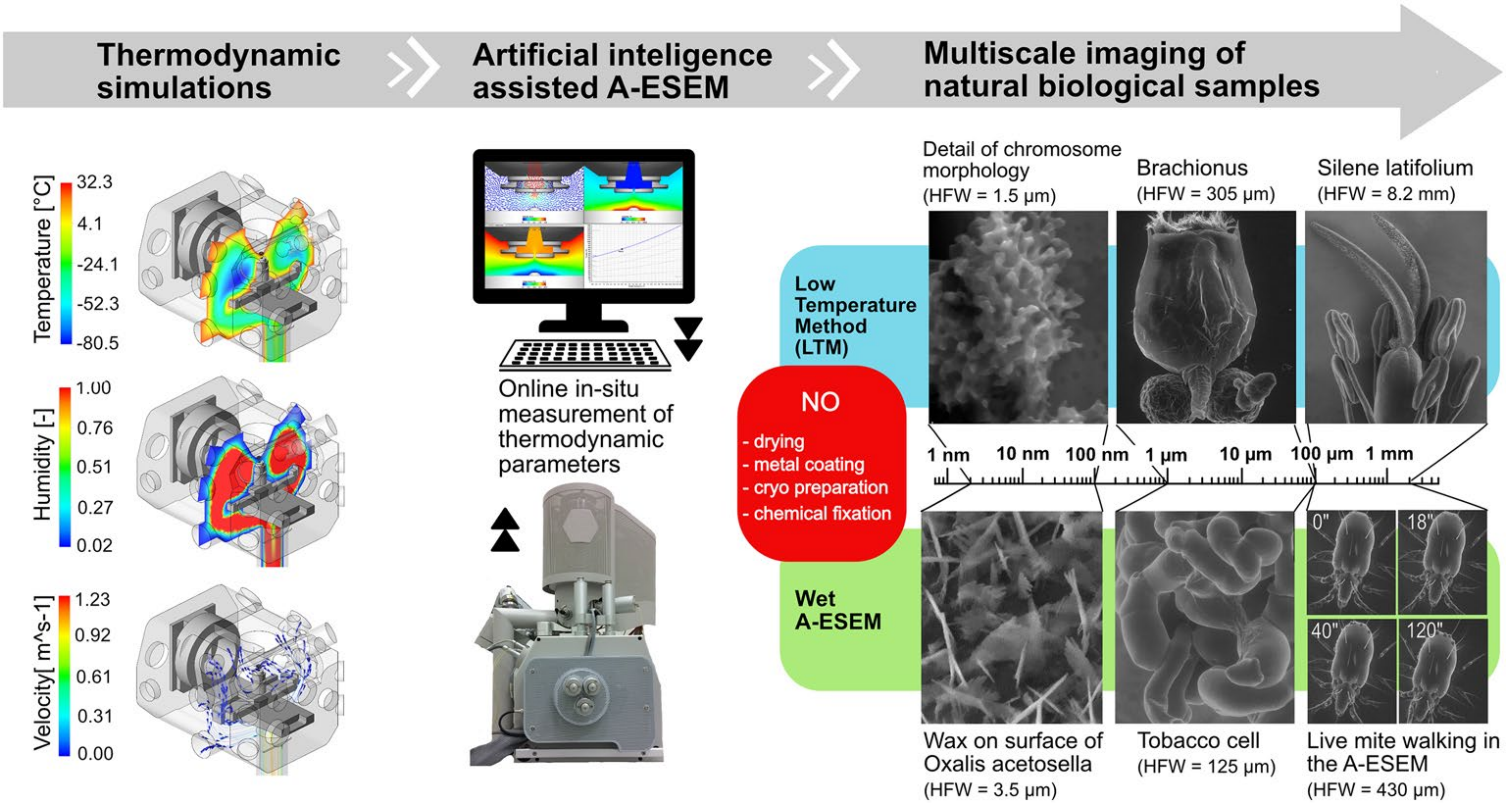
Artificial intelligence-assisted A-ESEM for macro- to nanoscopic imaging of biological samples in their native state.

A-ESEM is a novel, user-friendly method for complex in-situ physico-chemical analysis and macro- to nano-scale imaging of static or dynamically changing samples in their native state under environmentally compatible conditions, see Fig. 1.

We used a low temperature approach for in-situ handling and high-resolution imaging of extremely sensitive structures of condensed mitotic metaphase chromosomes of barley. It was based on a low-temperature method (LTM)^27^ and a newly developed artificial intelligence (AI) module for A-ESEM.

An artificial intelligence algorithm was developed using ThermoDynamic Simulator (TDS) software (https://www.numsolution.cz/en/products), based on a combination of computational fluid dynamics (CFD) numerical simulations in ANSYS CFX and the support vector regression method (ε-SVR). Results from CFD analyses were used as training data for the algorithm. TDS was used for the estimation of thermodynamic parameters involved in the initial settings of the sample environment protocol. Graphical outputs are visualized as contours placed in the vertical axis of the sample. The correct contour is selected using machine learning based on user specified input values. Based on TDS results, measurements of thermodynamic parameters were performed to obtain data for simulation in the Ansys CFX software aiming at optimizing working parameters of the LTM.

**Figure 2:**
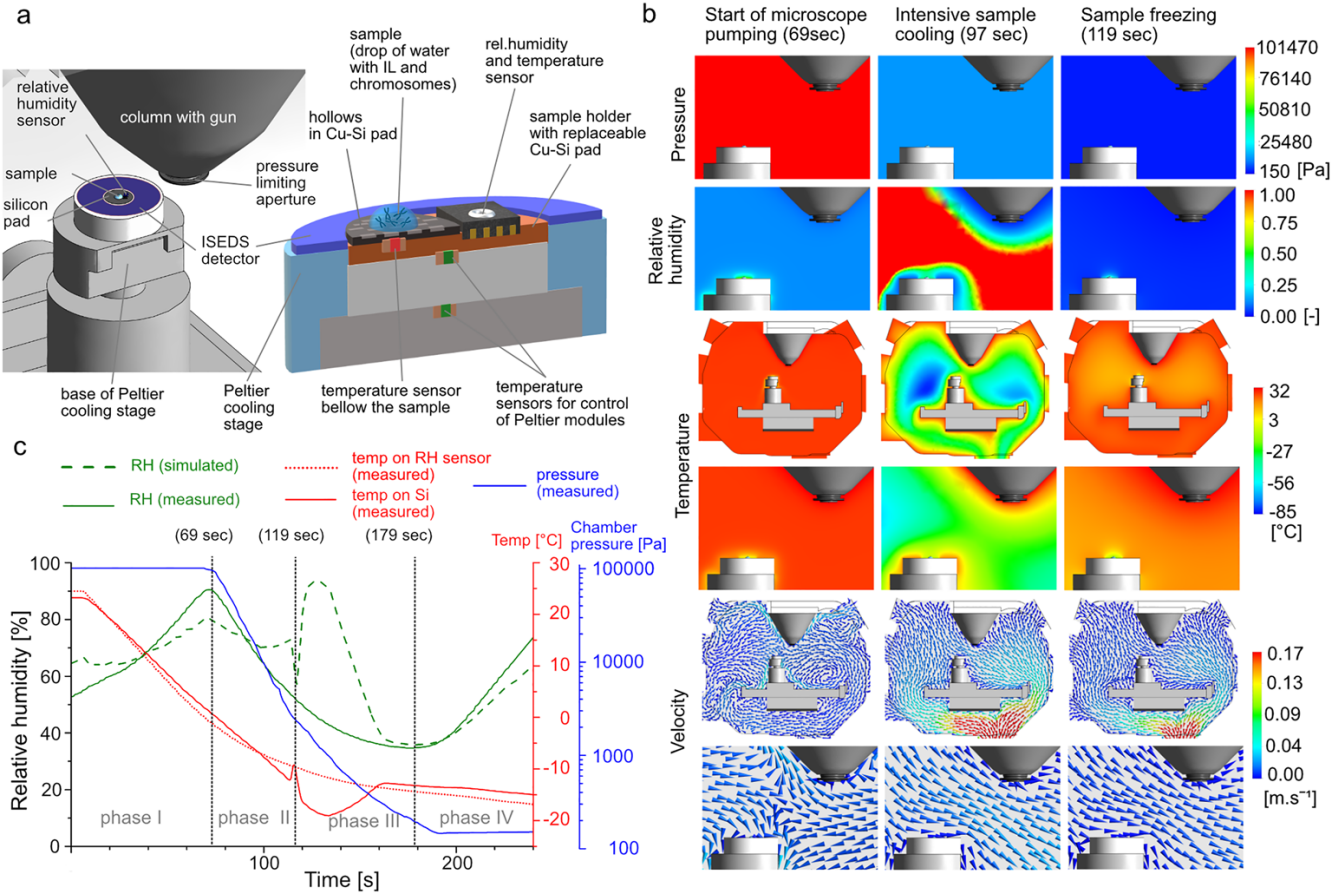
Spatial visualization of A-ESEM experimental set-up including. (**a**) A detailed cross-sectional view of the top of the cooled sample holder. a, the position of the sensors, and a drop of solution containing chromosomes placed on a Si pad with an etched checkerboard pattern. (**b**) Time dependences of measured and simulated thermodynamic parameters during individual phases of the LTM sample handling. (**c**) Visualization of the distribution of thermodynamic parameters and gas flow direction/velocity in the specimen chamber during processing using the LTM.

Environmental parameters for transient analysis were monitored in-situ using a calibrated temperature sensor placed just below a replaceable Cu-Si pad (Fig. 2a), humidity micro-sensors placed in the Cu base of the Cu-Si pad at the same level as the sample (Fig. 2a), and two gas pressure capacitance gauges in the wall of the specimen chamber. The sensor data were processed online using in-house software with a graphical interface to determine the dependence of total pressure, water vapor partial pressure and saturated water vapor pressure on the temperature and time at different points in the specimen chamber. The measurements obtained, in particular RH just above the sample surface, were used as boundary conditions for transient analysis of thermodynamic conditions during the LTM^28^. Significant changes in temperature when applying LTM can only be measured using the temperature sensor placed directly under the drop containing chromosomes (Fig. 2a,c; red line vs. doted red line). Therefore, the data from the RH sensor can only be used as one of the boundary conditions for the simulations. Artificial intelligence-based simulations are the only way to reliably deduce humidity changes close to the drop (Fig. 2c; green line vs. dashed green line).

Prior to the LTM, the specimen chamber was sufficiently hydrated through a series of purge flood cycles until the RH reached the required initial level of 65% (Fig. 2c; green line)^29^. Sample preparation for A-ESEM observation was kept to a minimum, with only the application of a highly diluted ionic liquid (IL). Since the isolated mitotic chromosomes were essentially in a native state (without harsh chemical treatments, post-fixation with osmium or drying) the IL could sufficiently permeate the sample. The chromosomes are sensitive to the environment in which they are stored, therefore the IL exposure time was minimized and no pre-incubation was applied. The ability of ILs to remain liquid in the supercooled state^30^ is ideal for the LTM, and the increased electrical conductivity of the surface covered by a very thin IL layer allows imaging of non-coated chromosomes (Fig. 4) under significantly lower gas pressure as compared to conventional ESEM. Increased thermal conductivity also allows for more rapid sample cooling. Very thin IL layer also helps to scavenge free radicals and thus to reduce radiation damage to the sample during low-dose imaging, reduces water evaporation from the sample, and does not visibly distort the natural nanostructure of the surface.

Before the observation, chromosome samples were equilibrated in-situ following the LTM process directly in the A-ESEM specimen chamber, on Cu-Si pad of the Peltier cooling stage^31^. To ensure optimal velocity and direction of gas flow around the sample, including underneath, and to minimize mechanical damage to the sample from the liquid–gas interface during drop evaporation, the sample was laid on Si with an etched checkerboard pattern. A matrix of 10 × 2 μm^2^ square holes with a spacing of 10 μm was etched onto the Si surface according to a pattern created by electron beam lithography. The shape of the pattern was designed based on experimental results and made it possible to image the very fine protrusion morphology on the chromosome surface (Fig. 4). The shape and dimension of the pattern, as well as its positive effect on the preservation of fine morphology was the outcome of a series of experiments. A series of regular depressions/thinning of the Si pad most probably causes it to be better cooled at the thinning points and disrupts the homogeneity of gas flow along the surface of the SI pad and around the sample. The sample is thus well surrounded by the gas and therefore its very fine morphology is preserved. The fine fibers on the sample surface are fluffed by the gas flow, they are not pressed towards the surface.

The application of the LTM can be described in four phases (Fig. 2c). In phase I (up to 69 s), the chromosome samples were pre-cooled from 25 to 0.5 °C (at a rate of 18.3 °Cm^−1^) in the closed specimen chamber under atmospheric pressure. Preferential evaporation of water previously condensed on the base of the Peltier cooling stage increases RH (green lines) and protects the sample from drying during the continuous temperature decrease (red line) of the Cu-Si pad. The sample is still fully covered with the liquid and the volume change of the drop is negligible. When the sample temperature of 0.5 °C is reached, phase II starts and the gas from the specimen chamber starts to be pumped out (Fig. 2c; 69 s). The key factor in the successful use of the LTM is a synchronized decrease in sample temperature and gas pressure with respect to the initial humidity in the specimen chamber^27^. The temperature decrease is accelerated both by the use of IL and by placing the sample near the region with the lowest temperature (down to − 85 °C) in the specimen chamber (Fig. 2b; Temperature—97 s). Here, the sample is intensively cooled by very cold gas flow (Fig. 2b; Velocity—97 s) until the end of phase II. At this stage, gas is pumped out at a rate of 2000 Pa/s and the evaporation of water from the droplet is very intensive (Fig. 2c; green dashed line). Under these conditions, the supercooled drop immediately freezes at − 14 °C (Fig. 2c; 119 s—start of phase III).

Higher the degree of supercooling, higher the rate of ice nucleation and the faster the effective rate of freezing. This results in a high number of small crystals of ice^32^. The water is continuously sublimated and exchanged for the ionic liquid. Sample freezing is accompanied by the release of the heat of crystallization (Fig. 2c; red line, small peak at 119 s). Sublimation of residual ice then occurs, indicated by the increase in simulated RH above the sample surface, up to 94%, and a decrease of sample temperature to − 19 °C. Finally, the thickness of the liquid layer on the sample is reduced, revealing the nanostructure of the sample covered only by an ultra-thin IL layer, not visible in the image. As a result of continuous gas pumping and the increase in sample temperature, RH decreases below 40% in 179 s (start of the phase IV). By opening the valve of the hydration system (185 s) to stabilize the gas pressure in the specimen chamber to 150 Pa (final value for imaging) RH increases to more than 60% at the end of the phase IV. The raise in pressure increases the probability of electron-induced ionization of gas molecules, which in turn increases the detection efficiency of the high-efficiency ionization secondary electron detector with electrostatic separator (ISEDS)^33^. When phase IV is completed, the sample is moved to the optical axis of the microscope and A-ESEM observation may start. The observation parameters were: electron beam energy 10 keV, beam current 10 pA, dwell time 5 µs. Due to the increased temperature of gas in close vicinity to the pole piece of the objective (Fig. 2a left, 2b in the middle) and thermal effects of the electron beam, any residual ice sublimates from the sample surface and so the study of very fine nanomorphology of chromosome surfaces is possible.

**Figure 3:**
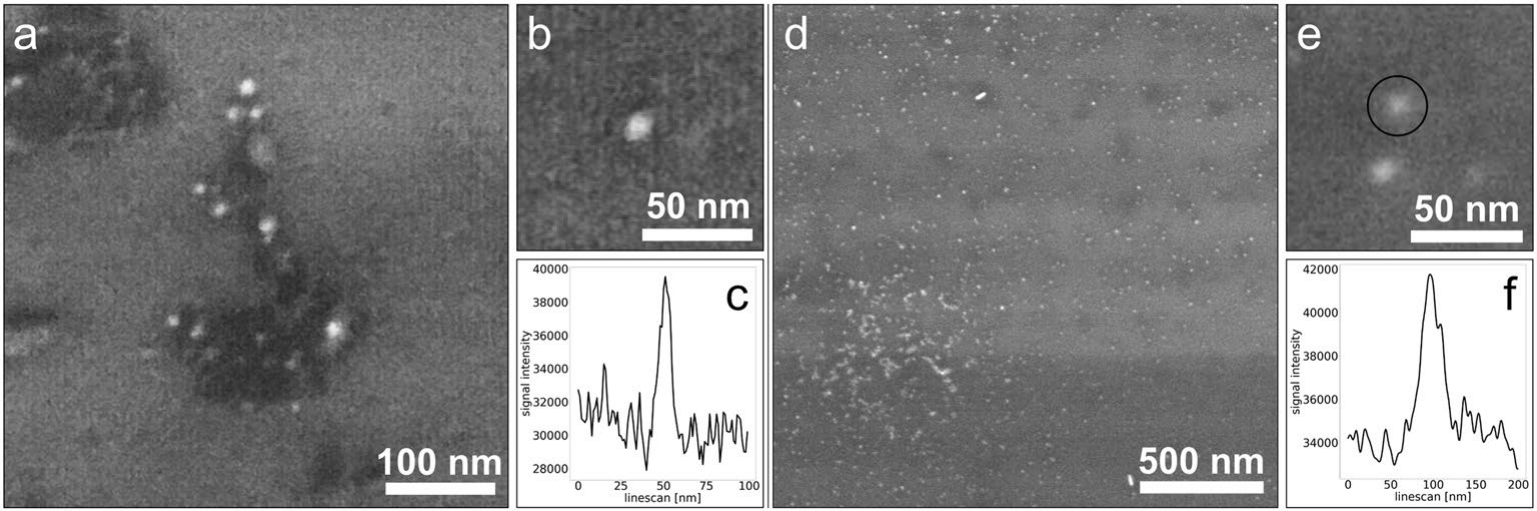
Spatial resolution measured under chromosome observation conditions using the A-ESEM. (**a**) Alexa Fluor® 546-FluoroNanogold™ nanoparticles enlarged to a final size of about 10 nm after application of the GoldEnhanceTM plus mixture, immunostained with tobacco cell plasma membrane proteins. (**b,c**) Detail image of the measured gold nanoparticle from (**a**) and its, line-profile over the gold nanoparticle. (**d**) 5 nm gold nanoparticles on silicon substrate. (**e,f**) A detail image of the measured gold nanoparticles from (**d**) and its line-profile over the gold nanoparticle within the black circle.

To verify the resolution reached with the A-ESEM under the conditions of chromosome observation we used a Alexa Fluor® 546-FluoroNanogoldTM nanoparticles immunostained with auxin efflux carriers within nanodomains of the plasma membrane of tobacco cells, see Fig. 3a,b and 5 nm gold nanoparticles on silicon substrate, see Fig. 3d,e. A detailed view of gold nanoparticles is shown in Fig. 3b,e. The line-profile over an individual gold nanoparticles used to measure resolution are shown in Fig. 3c,f. The measurements confirmed a resolution of 3.8 nm ± 0.6 nm from Fig. 3a,b and a resolution of 4.4 nm ± 0.9 nm from Fig. 3d,e (pixel size 0.4 nm).

### Chromosome observation

**Figure 4:**
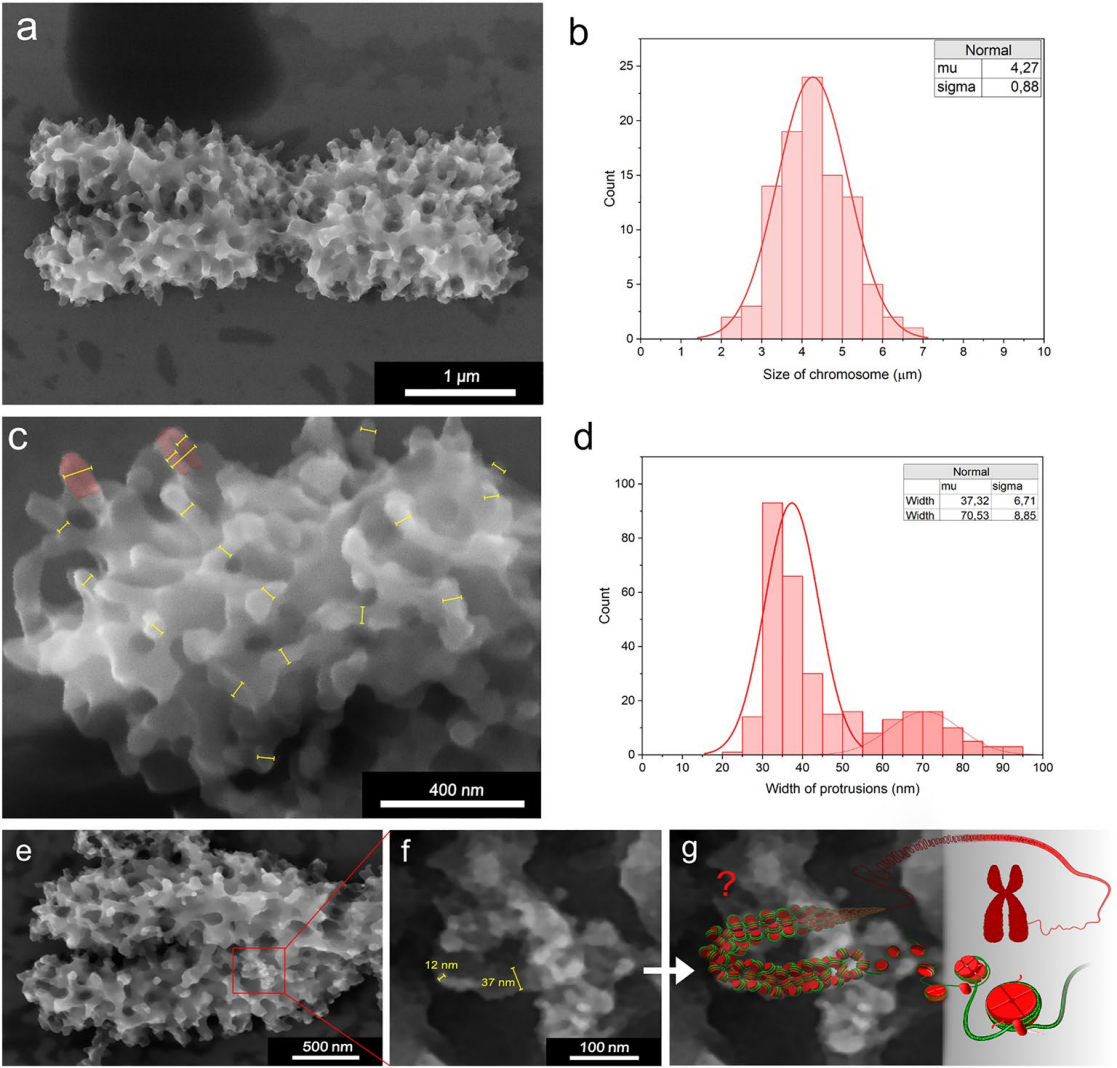
Barley (*Hordem vulgare*) mitotic metaphase chromosomes observed by A-ESEM, secondary electron detector. (**a**) Overview of a chromosome with protrusions covering it’s the entire body, including centromeric region, top view. (**b**) Histogram of chromosome length distributions as determined using A-ESEM (95 measurements). (**c**) Detailed view of the protrusions on the terminal telomeric chromosome region, with the sizes of the protrusions indicated (yellow bars). (**d**) Histogram of the protrusion widths (183 measurements). (**e**) Close-up of a chromosome region showing ~ 12 nm features, which may represent nucleosome fibers. (**f**,**g**) The ~ 12 nm features form ~ 37 nm structures (yellow bars), whose molecular composition is not clear (see the text for more details).

In contrast to previous SEM studies and conventional ESEM observations^6^, our approach involves greatly simplified preparation of chromosome samples to preserve their nanostructure. The only chemical treatment used was a mild formaldehyde fixation of root tips from which the chromosomes were released by mechanical homogenization and afterwards purified by flow cytometric sorting (Fig. 4a). No chemical fixation or heavy metal staining was used to prepare chromosome samples for imaging. High detection efficiency and SE/BSE ratio of the ISEDS^33^ also eliminated the need for additional contrast enhancement with gold nanoparticles or quantum dots. Due to the extremely low initial concentration of the IL in water (0.01% see text bellow; 100 times lower than the lowest concentration tested for wet cell imaging in SEM^34^ and its biocompatibility^35^ we don’t expect any morphological changes of the chromosome surface caused by the IL coverage.

As A-ESEM visualizes topography of chromosomes in a native state, our observations should not suffer from preparatory and observation artefacts, which are inherent to conventional SEM^36^ and cryoSEM^37^. The average length of A-ESEM imaged barley chromosomes measured using Scandium software was 4.3 µm (n = 95) (Fig. 4b), which was 20% less than the length of 5.3 µm (n = 200) as determined using fluorescence microscopy (data not shown). The difference could be due to longitudinal extension of chromosomes during drying on a glass slide prior to conventional fluorescence microscopy.

A-ESEM revealed chromosome surface studded with numerous protrusions covering densely the chromosome surface (Fig. 4c). The mean width of the protrusions was 37 nm (n = 183) (Fig. 4d); their nature is not clear. A close up of some chromosome regions indicated that ~ 37 nm structures are formed from ~ 12 nm features, which may represent nucleosomes (Fig. 4e–g). As the formation of 30 nm solenoid has not been confirmed in vivo^38^, the ~ 37 nm structures may represent nucleosome clutches^39^. We also observed protrusions with a mean width of 70.5 nm (n = 111), which could represent two structures fused together. Such organization of chromosome surface has not been observed before and this observation contributes to the efforts aiming at unravelling the organization and function of the perichromosomal layer^40^.

**Figure 5:**
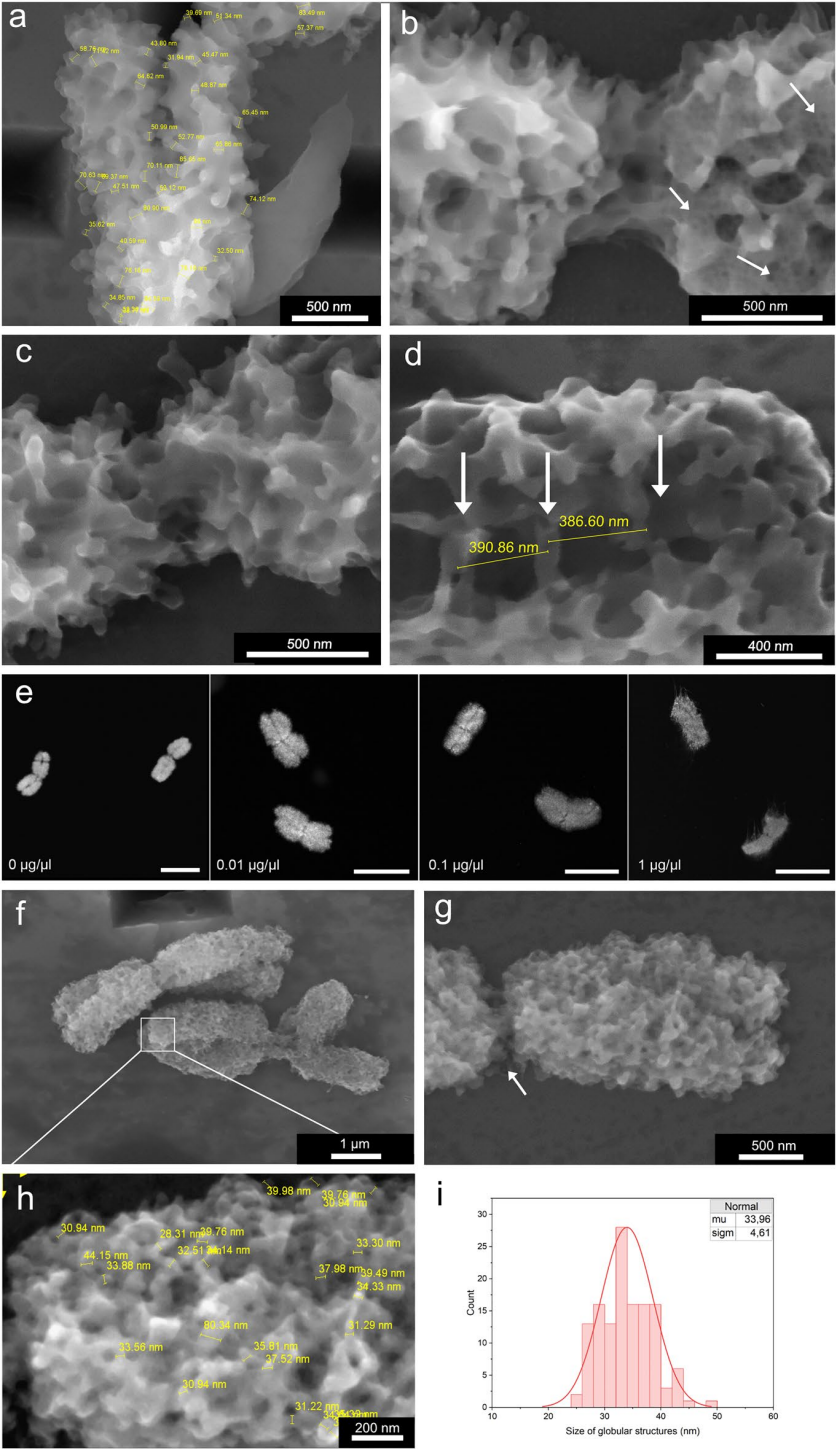
The effect of chromosomes post-fixation and prolonged exposure to electron beam. (**a**) Protrusions merge together so that their predominant size is ~ 60 nm (yellow bars) after post-fixation with 2% formaldehyde. Moreover, chromosome surface is smoother, with fewer distinct protrusions compared to non-treated chromosomes. (**b**) Radiation damage caused by prolonged exposure to electron beam. The left side of the chromosome was scanned only once, while the right side was scanned multiple times. White arrows point to regions where artificial cavities are formed. (**c**) Detail of the centromeric region, side view. (**d**) Bridges between sister chromatids with regular 390 nm spacing (yellow bars), (**e**) Fluorescence microscopy of chromosomes treated with RNase. Chromosomes were treated with RNase A at various concentrations for 30 min at 37 °C and the reaction was stopped by the addition of RNase inhibitor. Chromosomes were then stained using DAPI and observed by fluorescence microscopy. Increasing concentration of the enzyme disrupted the structure of chromosomes. As control, the chromosomes were incubated in RNase buffer. Scale bars represent 5 µm. (**f**,**g**) Chromosomes treated with RNase A and observed by A-ESEM exhibit changed chromosome surface organization with the absence of surface protrusions, missing interchromatid bridges and exposed parallel fibrils in the centromeric region (white arrow). (**h)** Measurement of globular structures that become apparent after RNase treatment (yellow bars). (**i**) Histogram of sizes of globular structures from Fig. h (105 measurements). A-ESEM images were obtained using the ISEDS.

Post-fixation of flow-sorted chromosomes with 2% (v/v) formaldehyde for 20 min at 5 °C resulted in a dramatic decrease in the number of the 30 nm protrusions and increased the number of double sized protrusions. Moreover, the surface of post-fixed chromosomes was smoother as compared to non-treated ones (Fig. 4a vs. Fig. 5a). The altered surface organization was clearly an artifact due to the post-fixation and this observation indicates the importance of avoiding any chemical treatment of isolated chromosomes to observe their topography in as close to a natural state as possible. Such treatments, however, are integral part of protocols used to observe chromosomes using SEM^25^.

A prolonged exposure to electron beam resulted in the observation of chromatin cavities (Fig. 5b). The presence of chromatin cavities was reported previously after the observation of mitotic chromosomes by SEM^20,40^. However, the cavities were not observed when ionic liquid preparatory method was used for SEM^20^. The absence of larger dark spots in non-treated chromosomes representing void space below the chromosome surface in this work indicates that the earlier observations of cavities were most probably due to preparatory artefacts. Given that our approach does not rely on critical point drying and on ionic liquid preparatory steps, it is suited to clarify the presence of chromatin cavities. The observation using A-ESEM suggested that condensed mitotic chromosomes are free of hollow spaces.

Contrary to the published SEM images of barley chromosomes^12,41,42^ our observation using A-ESEM did not reveal parallel fibrils in centromeric regions. The chromosome surface of this region was similar to the remaining parts of chromosome body, albeit with noticeable hinge and a decreased number of protrusions (Fig. 5c). An interesting feature were the bridges linking sister chromatids **(**Fig. 5d). The distance between the adjacent bridges was 390 nm, which compares well with 400 nm estimated earlier for human and pig chromosomes^43^ using a different method^44^. However, given the high compaction and the ~ 2 µm size of the mitotic chromosome arms, we observed correspondingly smaller number of bridges. Future study of molecular composition of the bridges should reveal their molecular composition and role in sister chromatid cohesion. Our observations indicate that they are part of the perichromosomal layer and free of DNA as they disappeared after a mild RNase treatment (see below).

A published model of hierarchical structure of perichromosomal layer suggests that its intermediate layer comprising mainly pre-ribosomal RNAs, plays an important role in binding proteins on chromosome surface and the formation of chromosome cover^24^. A removal of the RNA layer was reported to cause a loss of nucleolar proteins from human chromosome surface^24^. To confirm the role of RNA, we treated barley chromosomes with 0.01 µg µl^−1^ RNase for 30 min at 37 °C. This treatment caused only a minor swelling of chromosomes, but their overall shape remained unchanged as observed by fluorescence microscopy (Fig. 5e). However, A-ESEM revealed significant differences from the untreated chromosomes. The regular pattern of the 30- and 60 nm protrusions was no longer visible, chromosome surface appeared levelled and with globular structures (Fig. 5f,g). This pattern of chromosome surface organization was observed earlier by SEM^44,45^. A diameter of the globular structures ranged from 25.8 to 43.7 nm (n = 105) (Fig. 5h,i), and their size was thus smaller as compared to the protrusions in non-treated chromosomes. The marked change in the surface organization may be explained by the elimination of RNA and proteins from the perichromosomal layer, e.g., also from the protrusions. Moreover, RNase treatment exposed parallel fibers in centromeric region (Fig. 5g) and the bridges between sister chromatids could no longer be detected. According to Booth and Earnshaw the chromosome periphery, which was most probably lost after the RNase treatment, has a thickness of 87–150 nm^22^. However, we could not confirm this value due to chromosome swelling after RNase treatment.

## Conclusions

This study demonstrates the power of A-ESEM for imaging sensitive biological objects at nanometer resolution, eliminating a majority of preparatory and observation artefacts inherent to other types of SEM^36^. Spatial resolution measurements using 5 nm gold nanoparticles on a silicon substrate and immunostained gold nanoparticles in plasma membrane of tobacco cells confirm the resolution at the lower nm units, even at elevated gas pressure. Thus, our result confirms Danilatos’ earlier claim that under oligo-scattering conditions the resolution limit for ESEM can be the same as for SEM^46^. However, the contrast in the ESEM is reduced by the scattering of the electron beam in the gas. Under specific conditions for chromosome observation, the resolution of 3.8 ± 0.6 nm (pixel size 0.4 nm) was estimated (see Fig. 3a).

The observation of mitotic chromosomes using A-ESEM permitted visualization of chromosome topology at nanometer resolution and in a state as close to natural as possible. This was achieved by avoiding chemical fixation, heavy metal staining and coating, drying; the samples were not frozen for the observation in vacuum. Thanks to this, new insights into the topology of mitotic chromosomes were obtained. The new approach revealed that the surface of mitotic chromosomes is studded with numerous protrusions with the mean size of ~ 30 nm. Such organization of chromosome surface has not been observed before. Our observations support the model of hierarchical organization of the perichromosomal layer and the role of pre-ribosomal RNAs in its establishment, as well as the presence of regularly spaced bridges linking sister chromatids that were discovered recently^20,40^. Moreover, it is highly likely that our method enabled visualization of nucleosome fiber in its native state. The results provide an important step forward in the efforts to unravel the topography of condensed mitotic chromosomes that play a crucial role in faithful transmission of genetic information. A possibility to couple A-ESEM with other imaging techniques, including light microscopy^47^, will allow imaging and functional analysis of not only mitotic chromosomes but also other biological objects in their native state.

## Methods

### Isolation of condensed mitotic metaphase chromosomes

Seeds of barley (*Hordeum vulgare L.*) cultivar Morex (grown in Centre of Plant Structural and Functional Genomics, Olomouc, Czech Republic) were germinated, cell cycle in meristem root tip cells was synchronized with hydroxyurea and dividing cells were accumulated at metaphase with amiprophos-methyl^48^. The roots were then mildly fixed with 2% (v/v) formaldehyde in Tris buffer (10 mM Tris, 10 mM EDTA, 100 mM NaCl) for 15 min at 5 °C and then washed three times with Tris buffer for 5 min. Terminal 1–2 mm root tips were cut off, collected in LB01 buffer^49^ and homogenized with Polytron-PT1000 (Kinematica AG, Littau, Switzerland) at 15,000 rpm for 13 s. The homogenate was filtered through 50 µm mesh and subsequently through 20 µm nylon mesh into a 5 ml polystyrene tube. DNA of chromosomes in the suspension was fluorescently stained using DAPI and the samples were analyzed using a BD FACSAria SORP flow cytometer and sorter (BD, San Jose, CA). Initial gating was set on a dotplot DAPI-A vs FSC-A, and the subsequent final sorting gate was set on a dotplot DAPI-A vs SSC-A. For each experiment, one million chromosomes were sorted into 2 ml LB01 in 15 ml low-bind Falcon tubes.

Flow-sorted chromosomes were pelleted at 850 g for 30 min at 4 °C in a centrifuge with a swing bucket rotor, the supernatant was removed except for 15 µl and pelleted chromosomes were gently resuspended. Then the chromosomes were diluted with 15 ml ice-cold 0.025% Triton X-100 (prepared in DNase-free and RNase-free sterile ddH_2_O) and pelleted again in the same conditions. The supernatant was removed except for 100 µl and pelleted chromosomes were gently resuspended. This was the starting material for direct imaging by fluorescence and environmental scanning electron microscopy as well as for RNase treatment.

For RNase treatment, chromosome samples were incubated at 37 °C for 30 min with 0.01 µgµl^−1^ RNase A (Sigma-Aldrich, St. Louis, Missouri, USA) and the reaction was stopped by adding 200 U of RiboLock RNase inhibitor (Thermo Fisher, Waltham, MA, USA). The RNase solution was titrated in order to select optimum concentration to keep chromosomal shape intact with minimal chromosome swelling. As a control for RNase treatment, the reaction was carried out under the same conditions in the absence of the enzyme, when the pipetted volume was substituted with ddH_2_O. For fluorescence microscopy observations, 3 µl of chromosomal suspensions were pipetted into 3 µl drop of PRINS buffer supplemented with 2.5% sucrose^50^ directly on a microscope slide, dried, and mounted with VectaShield medium (Vector Laboratories, CA, USA). Fluorescence microscopy images were captured using Zeiss Axio Imager Z2 (Carl Zeiss Microscopy, Oberkochen, Germany) and Isis Software (MetaSystems Grou, Inc., Newton, MA, USA).

### ThermoDynamic simulator

The core of the algorithm was built on machine learning, specifically on a support vector machine (SVM), which works via statistical learning theory^51^ and Wolfe’s dual programming theory. Compared to other learning algorithms, SVM is a robust approach that has wide applications in functional regression and pattern recognition^52^.

TDS program calculates the integral values of temperature, pressure and humidity in the vicinity of the sample depending on the real working distance of the sample. Two of the above-mentioned parameters (according to the choice of the microscope operator) are entered and then the last one is calculated. The program takes into the account the real geometry of the sample chamber, the saturated water vapor flow rate and the temperature gradient from the temperature sensor across the sample pad and the sample with respect to their heat capacity and the conductivity of the materials. Considering the TDS results, parameter measurements were performed for further optimization using Ansys CFX software (https://www.ansys.com/products/fluids/ansys-cfx). Thermodynamic parameters were monitored by calibrated temperature sensor PT 100 (Pico Technology, Cambridgeshire, UK), humidity micro-sensors SHT 35 (Sensirion, Staefa, CH) and gas pressure capacitance gauges CMR 371 and 372 coupled with controller TPG 366 (Pfeiffer Vacuum, Aßlar, D).

The transient analysis was carried out in Ansys CFX with time step 0.05 s and total simulated time 240 s. Heat transfer phenomena within the sample environment was simulated using a total energy model, which includes high-speed energy effects (in our case, a rapid pressure drop during pumpdown of the microscope chamber). Laminar flow without turbulent models was used and radiation was not considered. A solver scheme was set to high resolution and second order backward Euler with maximum 100 iterations per time step. Humid air with temperature-dependent thermophysical properties was used as the fluid material. TDS also allows to visualize graphical outputs in the form of contours placed in vertical axis of the sample. The correct contour is selected using machine learning based on user specified input values.

### Artificial intelligence assisted A-ESEM development and observation

An in-house modified scanning electron microscope Quanta 650 FEG (Thermo Fisher Scientific, Waltham, MA, USA), equipped with a new prototype Peltier cooling stage and an integrated high-efficiency ionization secondary electron detector with electrostatic separator (ISEDS)^33^, was used for high-resolution, low-dose imaging of chromosomes in their native state. The instrument modifications included a redesigned differentially pumped chamber in the objective lens, an improved vacuum control and pumping system and an in-house developed hydration system for control of the direction, intensity and temperature of water vapour flow.

The IL 2-Hydroxyethyl-trimethylammonium l-(+)-lactate [(C_2_H_4_OH)(CH_3_)_3_N]-[Lactate] ([HE3MA]LAC) (> 98% purity, Sigma Aldrich) was used without further purification. The [HE3MA]LAC was used at an initial concentration of 0.1g l^−1^ H_2_O. A 1 μl drop of aqueous [HE3MA]LAC solution was placed on the Cu-Si pad of the Peltier cooling stage and mixed with 1 μl of chromosome sample. Chromosomes are very sensitive to the environment in which they are stored, therefore the [HE3MA]LAC exposure time was minimized and no pre-incubation was applied.

The pattern on the Si surface was created by electron beam lithography (Vistec EBPG5000plusES, Raith, Dortmund, D).

To estimate spatial resolution of the A-ESEM, we used two types of samples: (1) 5 nm gold nanoparticles (Sigma-Aldrich/Merc Cat. No. 741949-25ML, stabilized suspension in citrate buffer) on silicon substrate and (2) Alexa Fluor® 546-FluoroNanogold™ nanoparticles immunostained with auxin efflux carriers within nanodomains of the plasma membrane of tobacco cells, for more details see our previous paper^47^. As the size of the gold particles on the secondary antibody is 1.4 nm, the GoldEnhanceTM EM plus mixture (Cat. No. 2114, Nanoprobes, Yaphank, NY, USA) was used to enlarge the Au nanoparticles to a size of approximately 10 nm. All of the above samples were imaged under the same conditions as the chromosomes. Spatial resolution was determined using the Line-profile over a selected nanoparticle of each type. The resolution was measured as half the width difference at 20% and 80% of the peak signal level. This procedure was performed separately for 5 nanoparticles, each on the Y axis, after applying a 0.1 pixel Gaussian filter using Affinity designer software.

### Additional information

The plant collection and use were in accordance with all the relevant guidelines.

## Data Availability

The datasets used and/or analysed during the current study available from the corresponding author on reasonable request.
